# Latest Trends in the Improvement of Measuring Methods and Equipment in the Area of NDT

**DOI:** 10.3390/s21217293

**Published:** 2021-11-02

**Authors:** Daria Wotzka, Michał Kozioł, Tomasz Boczar, Michał Kunicki, Łukasz Nagi

**Affiliations:** Faculty of Electrical Engineering Automatic Control and Informatics, Opole University of Technology, 45-758 Opole, Poland; m.koziol@po.edu.pl (M.K.); t.boczar@po.edu.pl (T.B.); m.kunicki@po.edu.pl (M.K.); l.nagi@po.edu.pl (Ł.N.)

The adequate assessment of key apparatus conditions is a hot topic in all branches of industry. Various on-line and off-line diagnostic methods are widely applied to provide early detections of any abnormality in exploitation. Furthermore, different sensors may also be applied to capture selected physical quantities that may be used to indicate the type of potential fault. The essential steps of the signal analysis regarding the technical condition assessment process may be listed as: signal measurement (using relevant sensors), processing, modelling, and classification. In the Special Issue entitled “Advances in Sensors and Sensing for Technical Condition Assessment and NDT”, we present the latest research in various areas of technology.

First, we present the outcomes of the teams of researchers who described the results of attempts to improve measurement methods and create devices to reduce errors and enable operations in real-time. In [1], authors deal with the area of high-current pulse measurement, particularly in cases where the current shunts that were used exhibited a limited frequency response. Pulse current values of the order of several kA, recorded with the shunt and Rogowski coil, indicate significant differences in the waveforms. Based on the theoretical and experimental research, the authors proposed a method that can be applied in real-time due to its simplicity. Their method, which greatly improves the accuracy of current wave measurement, uses a shunt circuit model created in ANSYS/Q3D to obtain the correct current wave from the solution of the ordinary differential equation (ODE). The article [2] addresses the issues related to linear displacement measurement systems. The authors presented a new method for the real-time compensation of geometric and thermal errors of an optical linear encoder. The algorithm of operation was designed based on the results of theoretical and experimental studies. A regression process was applied using a parametric function model under various ambient temperature dependences and a field programmable gate array (FPGA) for computation. Process quality and machine performance depend on the accuracy of the integrated linear encoder. The authors proposed a method that can reduce positioning error by as much as 98%. They point towards the need for further work related to other encoder designs and mounting methods. In [3], the authors deal with the area of bridge diagnostics and, in particular, the effect of radiation intensity on the stresses that occur in a five-span continuous prestressed-concrete box–girder bridge structure. The authors recorded solar radiation and surface temperatures, wind speeds, displacement, and strain for a period of 12 months and performed a correlation analysis, which showed that the most significant parameter affecting the tensile stress in the material is the lateral temperature gradient. The authors also performed finite-element analyses and developed a simplified numerical model that they plan to expand in the future. In [4], the authors present a Fizeau fiber interferometer to detect internal defects in aluminum plates using ultrasonography. The authors of [5] consider a combination of piezocomposite transducers and pulse-compression-processing methods to study the propagation of ultrasonic signals through steel pipes insulated with different materials. Using a combination of both methods, the SNR could be increased when measuring the signals reflected from insulated and cladded steel pipes. They prove that the application of ultrasound in the 100–400 kHz frequency range can be successfully applied for diagnostic purposes.

Artificial intelligence methods are being increasingly used in the field of diagnostics. In [6], the authors consider the electromagnetic non-destructive evaluation of conductive materials. They develop a new algorithm for eddy current testing (ECT), which uses wavelet processing and neural networks to study electromagnetic field fluctuations. They examine material samples made of austenitic stainless steel. They also focus on developing a method that is robust to disturbances and can reduce the error evaluation of material defect depth by 10%, even when the signal-to-noise ratio reaches 10 dB. In [7], the authors aim to improve the diagnosis of on-load tap changers (OLTC) using the acoustic emission method. They investigated the impact of the measuring device parameters on the accuracy of the classification of various OLTC defects. The authors applied time, frequency, and wavelet transformation methods to determine the features for the purpose of classification using various machine learning methods. In [8], the authors depict an automatic method to detect the model, type, and power of a distribution transformer based on the low-frequency noise emitted by the device. They applied machine learning and genetic algorithms for the classification of 16 different distribution transformers, achieving high accuracy.

Infrasound was also considered for the study of wind turbine noise. In [9], the authors looked at the issue of infrasound noise emitted by a 2 MW wind turbine. They described a measurement system that allows for the simultaneous measurement of noise at three locations, as well as signal processing methods, including wavelet coherence. The applied methods enabled determination of the differences and similarities in the noise levels that were registered around the turbine under consideration.

In the rest of the book, we include the results of work aiming to improve the measurement methods and advanced processing tools in various diagnostic areas. In [10], a methodology is presented for measuring and analyzing data to identify a tramway line’s axis system and support railway infrastructure management processes in planning and maintenance. The authors conducted experimental work using a global navigation satellite system (GNSS). Their algorithm uses multi-device data and repeated position recordings for identification of the main track directions of the tramway. They emphasized the assessment of the signal’s dispersion and repeatability using residuals in relation to the estimated track’s direction. In [11], the authors considered medical imaging methods that are capable of noninvasively detecting the compositions and variations of muscles. Their aim was to develop a method for the precise diagnosis of a specific muscular disease. They proposed a window-modulated compounding (WMC) Nakagami parameter ratio imaging and a time–Nakagami parameter ratio curve (TNRC) approach for the estimation of perfusion parameters, which can be used for the diagnosis of a specific muscle disease. Their methods provide reproducibility and robustness and a better sensitivity and tolerance of tissue clutters than conventional methods. Article [12] considers the application of classification methods in the field of joint diagnosis. The authors recorded Vibroarthrography signals, assigned to three stages of patella chondromalacia, osteoarthritis, and a healthy knee joint. The authors developed ten new features, calculated in the frequency domain, which were subjected to classification using ten different classification algorithms. The calculated Bhattacharyya coefficient values indicated an average of 9% better accuracy.

The final research area of diagnostics included in this Special Issue is monitoring the hatching process of eggs. The authors of [13] developed a prototype device, whose effectiveness was confirmed in verification studies. The advantage of the device is its ability to be used in industry for the fast and accurate detection of unfertilized eggs and dead-in-shell eggs on the ninth day of hatching. In this study, the authors used an image-processing method and a spectrophotometry technique in the 580 nm-wavelength LED range as the detection light source.
